# Curcumin mediates oxidative stress to play an anti-fibrotic role, focusing on liver, renal, myocardial and pulmonary fibrosis

**DOI:** 10.3389/fphar.2025.1636538

**Published:** 2025-11-21

**Authors:** Shishuang Yu, Jufang Pu, Ke Liu, Cuifang He, Feifei Yang, Ke Chen, Xiuli Yang, Yi Zhu, Jun Jiang, Maocai Luo, Xiao Liu, Chuantao Zhang, Yujiao He

**Affiliations:** 1 Department of Respiratory Medicine, Hospital of Chengdu University of Traditional Chinese Medicine, Chengdu, Sichuan, China; 2 Department of Chinese Internal Medicine, Chengdu Xinjin District Hospital of Traditional Chinese Medicine, Chengdu, China; 3 College of Acupuncture and Massage, Chengdu University of Traditional Chinese Medicine, Chengdu, Sichuan, China; 4 Department of Respiratory and Critical Care Medicine, Affiliated Fifth People’s Hospital of Chengdu University of Traditional Chinese Medicine, Chengdu, Sichuan, China; 5 Anti-infective Agent Creation Engineering Research Centre of Sichuan Province, School of Pharmacy, Sichuan Industrial Institute of Antibiotics, Chengdu University, Chengdu, China

**Keywords:** reactive oxygen species, reactive nitrogen species, curcuma, anti-fibrosis effects, traditional chinese medicine, signaling pathways

## Abstract

**Background:**

Curcumin is a natural polyphenolic compound that originates from turmeric (*Curcuma longa L., Linnaeus, Zingiberaceae*), a traditional medicinal herb. It is widely recognized for its strong antioxidant properties.

**Objective:**

This comprehensive review aims to delineate the recent progress in comprehending the role of curcumin in modulating oxidative stress and exerting an anti-fibrotic effect, with a particular focus on liver, renal, myocardial, and pulmonary fibrosis.

**Methods:**

A systematic review of the literature was conducted via the PubMed, Web of Science, Google Scholar, and China National Knowledge Infrastructure, covering 2000 until 2024. A systematic review identified studies examining curcumin’s regulation of oxidative stress pathways in therapeutic strategies for multiple fibrotic disorders, which were analyzed to synthesize current evidence.

**Results:**

In recent years, the application of curcumin for the clinical management of fibrotic diseases in a variety of clinical applications has been extensively investigated. Accumulating evidence suggests that curcumin can exert antifibrotic effects by ameliorating oxidative stress through the modulation of various signaling pathways such as regulating reactive oxygen species (ROS), nuclear factor erythroid-2-related factor 2 (NRF2), peroxisome proliferator-activated receptors (PPAR), transforming growth factor- β1 (TGF-β1). In this review, we investigate the pharmacokinetics of curcumin, the relationship between oxidative stress and the pathogenesis of fibrosis, and summarize the related studies of curcumin in the treatment of fibrotic diseases by regulating oxidative stress.

**Conclusion:**

This comprehensive review elucidates curcumin’s antifibrotic potential and explores its translational applications in developing novel therapeutic strategies to combat fibrotic pathologies, supported by mechanistic evidence that informs safer, more effective treatment paradigms.

## 1 Introduction

Fibrotic disease is a widespread disease that critically threatens public health, mainly including pulmonary fibrosis, liver fibrosis, renal fibrosis, myocardial fibrosis, peritoneal fibrosis, and other diseases. It has been widely reported to be characterized by high morbidity and mortality (Olson et al., 2007; Pottier et al., 2014). 45% of mortality in developed countries is related to cumulative impairment of tissue-specific function and final organ dysfunction due to fibrosis (Nanthakumar et al., 2015). It is noteworthy that with the acceleration of the aging of population, the above situation will become increasingly serious, and the morbidity and mortality rates will continue to increase (Redente et al., 2011; Kurundkar and Thannickal, 2016). Fibrosis is the procedure of aberrant aggregation of ECM after different types of tissues being damaged. It is a pathological stage of poor repair of all organs and tissues (Henderson et al., 2020; Panizo et al., 2021). Current treatment strategies for fibrotic diseases have many obstacles, including the lack of cell or tissue specificity of anti-fibrotic treatments, the occurrence of adverse events of anti-fibrotic drugs, and limited treatment options (Ramachandran and Henderson, 2016; Nastase et al., 2018; Lamb, 2021). Over the past decade, despite increasing research in the management of fibrotic diseases, its illness and death rate have continued to increase. Therefore, the development of anti-fibrotic therapeutic strategies holds substantial clinical importance. Traditional Chinese medicine (TCM) has a long history which has played a decisive status in the evolution of medicine in China for more than 2,000 years. Existing evidence shows that herbal medicine can serve as an important supplement and alternative method for anti-fibrotic drug treatment (Li et al., 2021; Li et al., 2022). It has the advantages of wide source, low toxicity, and structural diversity (Shan et al., 2019).

Turmeric (*Curcuma longa L., Linnaeus, Zingiberaceae*) is the rhizome of a perennial herbaceous plant of the ginger family, mainly distributed in southern and southwestern tropical Asia region (Aggarwal et al., 2007; Kocaadam and Şanlier, 2017). In TCM, turmeric was first documented in “New Materia Medica” (Hu Chenxia, 2008). It has the effects of invigorating blood circulation, and relieving depression (Qin et al., 2022). In clinical practice, turmeric is commonly used in a variety of herbal formulas to treat a wide array of diseases and body pathologies (Yang et al., 2019; Wu Yuhong and Liu, 2020; Zhengtao et al., 2022). Curcumin, a phenolic compound isolated from turmeric in modern times, is the main active ingredient responsible for the therapeutic effects of turmeric (Esatbeyoglu et al., 2012; Priyadarsini, 2014). Curcumin is often used as a pigment and spice in food, cosmetics, and textiles to dye and enhance flavor. It can also treat multi-system diseases (Aggarwal et al., 2007; Kocaadam and Şanlier, 2017; Qin et al., 2022), such as dermatologic diseases, infection, stress, and depression. Research on various diseases has proven that curcumin has anti-inflammatory, antioxidant, anti-cancer, antibacterial, anti-parasitic, anti-viral, and immune-modulating effects (Prasad et al., 2014; Kotha and Luthria, 2019; Zheng et al., 2020; Abd El-Hack et al., 2021; Luo et al., 2023). Different concentrations of curcumin have no obvious toxic effects on normal tissues and cells (Aggarwal and Harikumar, 2009). Therefore, curcumin is widely used in clinical research on various diseases.

**FIGURE 1:**
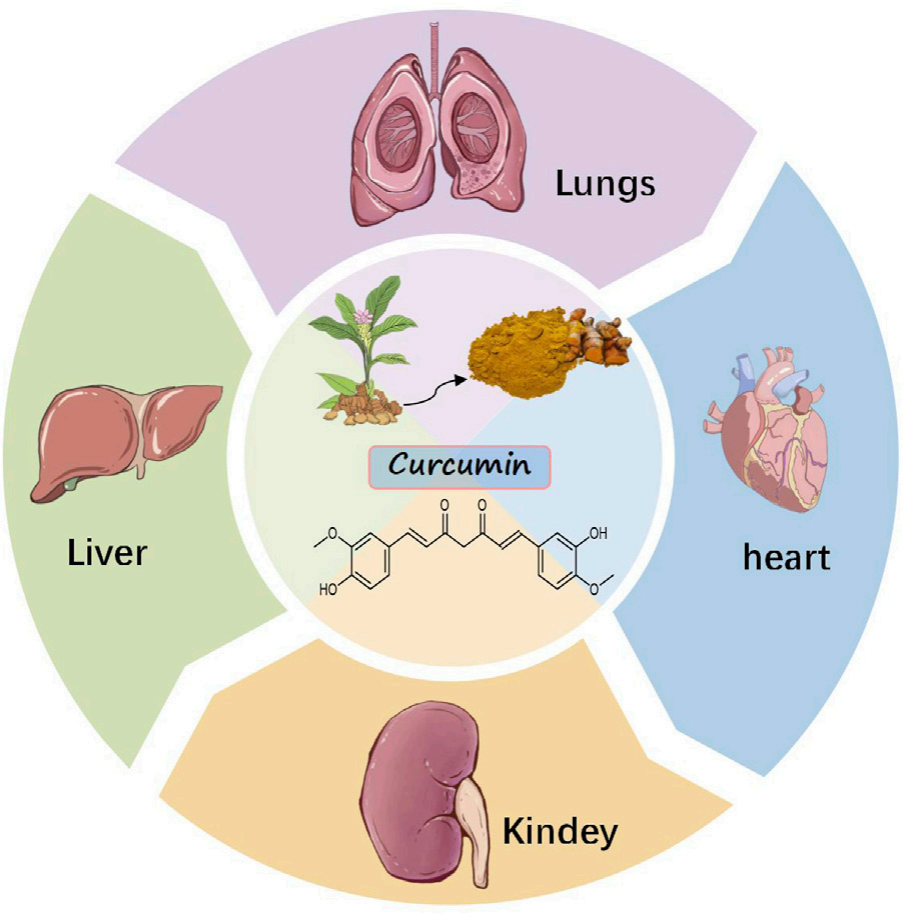
Antifibrotic effects of curcumin.

Curcumin, a lipophilic polyphenolic compound with low molecular mass, demonstrates significant therapeutic efficacy alongside a favorable safety profile. Numerous studies have proved the anti-fibrotic traits of curcumin, primarily in the lung, liver, kidney, and myocardial fibrosis (Kong et al., 2020; Sadoughi et al., 2021) (Figure 1). The mechanism may be related to inhibiting extracellular matrix (ECM) deposition, oxidative stress, and alveolar epithelial cells (AEC) cell apoptosis, reducing inflammatory response, and enhancing autophagy (She et al., 2018; Hernández-Aquino et al., 2020; Fathimath Muneesa et al., 2022; Cui et al., 2023). Among them, oxidative stress plays an important role in the initiation and development of fibrosis by damaging lipids, proteins, and DNA, inducing cell necrosis and apoptosis, amplifying inflammatory responses, stimulating the production of pro-fibrotic mediators, etc (Sánchez-Valle et al., 2012). This article reviews the mechanism of curcumin’s anti-fibrosis effects by inhibiting oxidative stress, to provide a reference for subsequent research.

## 2 Methods

A comprehensive online search of literature was conducted via Web of Science, Google Scholar, and China National Knowledge Infrastructure, covering 2000 until 2024. The ensuing key concepts were utilized: Curcumin, Turmeric Yellow, Curcumin Phytosome, Diferuloylmethane, Oxidative Stress, Antioxidative Stress, Oxidative Damage, Oxidative Stress Injury, Oxidative Injury, Oxidative Cleavage, Oxidative DNA Damage, ROS, Oxidative and Nitrosative Stress, Oxidative Nitrative Stress, Nitro-Oxidative Stress, Fibrosis, Cicatrix, Fibrosing, Hypertrophic, Keloid, Tissue Adhesions, Cirrhosis, fibrillation, fibration and fibering. A comprehensive examination was also conducted on the bibliographies of all the articles obtained from the search, aiming to incorporate pertinent literature.

## 3 Extraction, chemical structure, and physicochemical properties of curcumin

Curcumin serves as a natural compound sourced from Zingiberaceae plants, a vital group of medicinal plants (Shakeri et al., 2019). Generally speaking, curcumin can be extracted from the rhizomes or roots of ginger plants (Nan et al., 2023), which are customarily called turmeric (Curcuma) or turmeric in traditional Chinese medicine, respectively (Jianmin et al., 1983). An analysis of the rhizome or root extracts of ginger plants using thin-layer chromatography confirmed that the curcumin content in different plants of the same genus and even in rhizomes and roots of the same plant are significantly different (Jianmin et al., 1983). Qi et al. analyzed the curcumin content in the same species of ginger plants from different regions by HPLC and confirmed that the curcumin content was affected by the species of curcuma (Jianmin et al., 1983; Aidi and Wenyujin, 2002).

In 1953, Srinivasan determined the presence of curcumin and other components in turmeric by chromatography (Kocaadam and Şanlier, 2017). Most crude extracts prepared from turmeric mainly include curcumin (I), desoxymethylcurcumin (II), and dideoxymethylcurcumin (III) (Priyadarsini, 2014). The physicochemical properties of curcumin are presented in Table 1. It can be rapidly degraded when exposed to conditions including an alkaline pH environment (Dellali et al., 2021). Later, Chandrasekhara and Ravindranath et al. synthesized curcumin from vanillin and acetylacetone by condensation using the Pabon method with more than 99% content determined by the rose anthocyanin method (Ravindranath and Chandrasekhara, 1980; 1981a).

**TABLE 1:** Physicochemical properties of curcumin.

Content	Description
Name	Curcumin
CAS number	458-37-7
Molecular formula	C21H20O6
*In vitro* studies on the intestinal absorption of curcumin in rats	(1E,6E)-1,7-bis(4-hydroxy-3-methoxyphenyl)hepta-1,6-diene-3,5-dione
Canonical SMILES	COC1=C(C=CC(=C1)C=CC(=O)CC(=O)C=CC2=CC(=C(C=C2)O)OC)O
Molecular weight	368.37 g/mol
Topological Polar SurfaceArea	93.1Å²
Refractive Index	1.5118
Physical description	A crystalline solid
Color	Orange crystalline powder
Melting Point	183 ℃
Solubility	Insoluble in water; very soluble in ethanol, acetic acid
Density	0.9348 at 59°F (NTP, 1992) - Less dense than water; will float

## 4 Pharmacokinetics and toxicology of curcumin

Pharmacokinetics is the study of drug absorption, distribution, metabolism, and excretion, which helps us evaluate the properties of specific drugs and their application prospects. At present, there are a large number of literature studies on metabolic process of curcumin in rats, which are mainly administered by intraperitoneal injection, sublingual vein, oral administration, or the pharmacokinetics of curcumin in rats (Holder et al., 1978; Wahlström and Blennow, 1978; Ravindranath and Chandrasekhara, 1980; 1981a; b; Wang et al., 1997). Wahlstrom et al. reported that plasma levels and bile excretion measurements showed malabsorption of curcumin from the intestine in Sprague-Dawley rats at an oral dose of 1 g/kg (Wahlström and Blennow, 1978). Ravindranath et al. used radioactive tritium-labeled drugs to evaluate the tissue distribution of curcumin. The outcomes demonstrated that when Sprague-Dawley rats were given oral curcumin doses of 400 mg, 80 mg, and 10 mg, the percentage of curcumin absorption did not change. This demonstrates a dose-dependent limitation in curcumin absorption bioavailability (Ravindranath and Chandrasekhara, 1981b). Once curcumin is absorbed, it undergoes conjugation effects at different tissue sites. Research has found that curcumin-glucuronide, dihydrocurcumin-glucuronide, tetrahydrocurcumin-glucuronide and tetrahydrocurcumin are the main metabolites of curcumin in the body (Pan et al., 1999; Hoehle et al., 2006). Studies have shown that when rats are administered 100 mg/kg by intraperitoneal injection, curcumin levels are highest in the liver, followed by the spleen (Pan et al., 1999). However, in another human clinical study, 12 patients with colorectal cancer liver metastases received curcumin about 450–3,600 mg of per day 1 week before surgery. At the end, no curcumin was detected in the liver tissue, and only a small amount of reduced turmeric metabolites was detected (Garcea et al., 2004). Moreover, the rate at which curcumin is eliminated from the body serves as another crucial determinant of its biological impacts. Studies have shown that when Sprague-Dawley rats take an oral dose of 1 g/kg of curcumin, 75% of it is defecated in the feces, while the content of curcumin in the urine is negligible (Wahlström and Blennow, 1978). In another study, subsequent to the oral administration of a 400 mg dose of curcumin to rats, no traces of curcumin were discernible in their urinary samples (Ravindranath and Chandrasekhara, 1980). Metabolic studies using radiolabeled curcumin in rats, when administered at 400 mg/animal, about 40% of the unaltered form of curcumin was found in the feces (Ravindranath and Chandrasekhara, 1981b). Regardless of the dosage, the urinary excretion of curcumin is very low. The temporal parameters for the absorption and clearance phases of curcumin (2 g/kg) after oral ingestion in rats are 0.31 ± 0.07 and 1.7 ± 0.5 h respectively (Anand et al., 2007). One clinical study found that patients with advanced colorectal cancer who took turmeric extract orally everyday for 4 months contained 36–180 mg of curcumin and had neither curcumin nor curcuminoids in their urine metabolites (Sharma et al., 2001). Overall study results indicate that oral curcumin has low absorption and rapid clearance (Mirzaei et al., 2017). The above characteristics such as low bioavailability, short plasma half-life, low plasma concentration, and poor oral absorption have seriously limited the clinical development of curcumin (Tomeh et al., 2019; Hao et al., 2023). The study stated that when administered at the maximum recommended dose of 5 g/Kg, there was no obvious toxic effect on Sprague-Dawley rats (Wahlström and Blennow, 1978). Similarly, Aggarwal et al. studied the acute toxicological damage of curcumin-essential oil complex (CEC), an available biological agent, in rats and mice at the uppermost advised dose of 5,000 mg/kg. In contrast to the control group, these animals also showed no symptoms, toxicity, or death (Aggarwal et al., 2016). Findings from Phase I clinical trials involving curcumin indicate its safety in humans, even when administered at elevated doses (12 g/day) (Anand et al., 2007). However, more reliable research is needed to prove whether there is any difference in the toxic damage to internal organs and cells between animal and human bioavailable preparations.

## 5 Oxidative stress and fibrotic diseases

The process of fibrosis involves excessive deposition of ECM and remodeling of the injured site, leading to the restoration of unnecessary connective tissue and organ dysfunction during the repair process (Lurje et al., 2023). It may be present in virtually all organs of the human organism, such as the liver, kidneys, heart, lungs, and skin. Repeated injury is a general characteristic of fibrotic diseases. There are different causes, such as chronic virus infection, autoimmune, chronic ischemia process, or toxicity (including nicotine, alcohol, drugs, or radiation) (Distler et al., 2019). Fibrosis begins with the triggering of parenchymal cell damage or death and is usually repetitive or persistent. The subsequent repair process is mediated by regulatory processes such as damage recognition, rapid myeloid attraction and fibroblast activation. However, they lack the capacity to restore physiological equilibrium (Lurje et al., 2023). These fibrosis are typically characterized by the chronic inflammatory response coupled with alterations in the invasive immune cell infiltrate (Guillot and Tacke, 2019). Endogenous early-phase proinflammatory mediators including IL-1, IL-6, TNF, and TGF-β derived from macrophages, tissue fibroblasts, and resident stromal populations drive the differentiation of IL-17-producing effector cells. IL-17A promotes tissue damage through the production of ROS that enhances the neutrophilic response and meanwhile increases the expression of TGF-β receptors on fibroblasts, thereby promoting ECM production in the TGF-β response (Henderson et al., 2020). Immune cell subsets further drive fibrotic progression through the release of angiopoietic and fibrogenic mediators that act on resident fibroblasts and vascular endothelial cells, thereby inducing pathological neovascularization, and promoting tissue damage by secreting matrix metalloproteinases and ROS (Pardo et al., 2016; Bartneck et al., 2019; Sutti et al., 2019).

**FIGURE 2:**
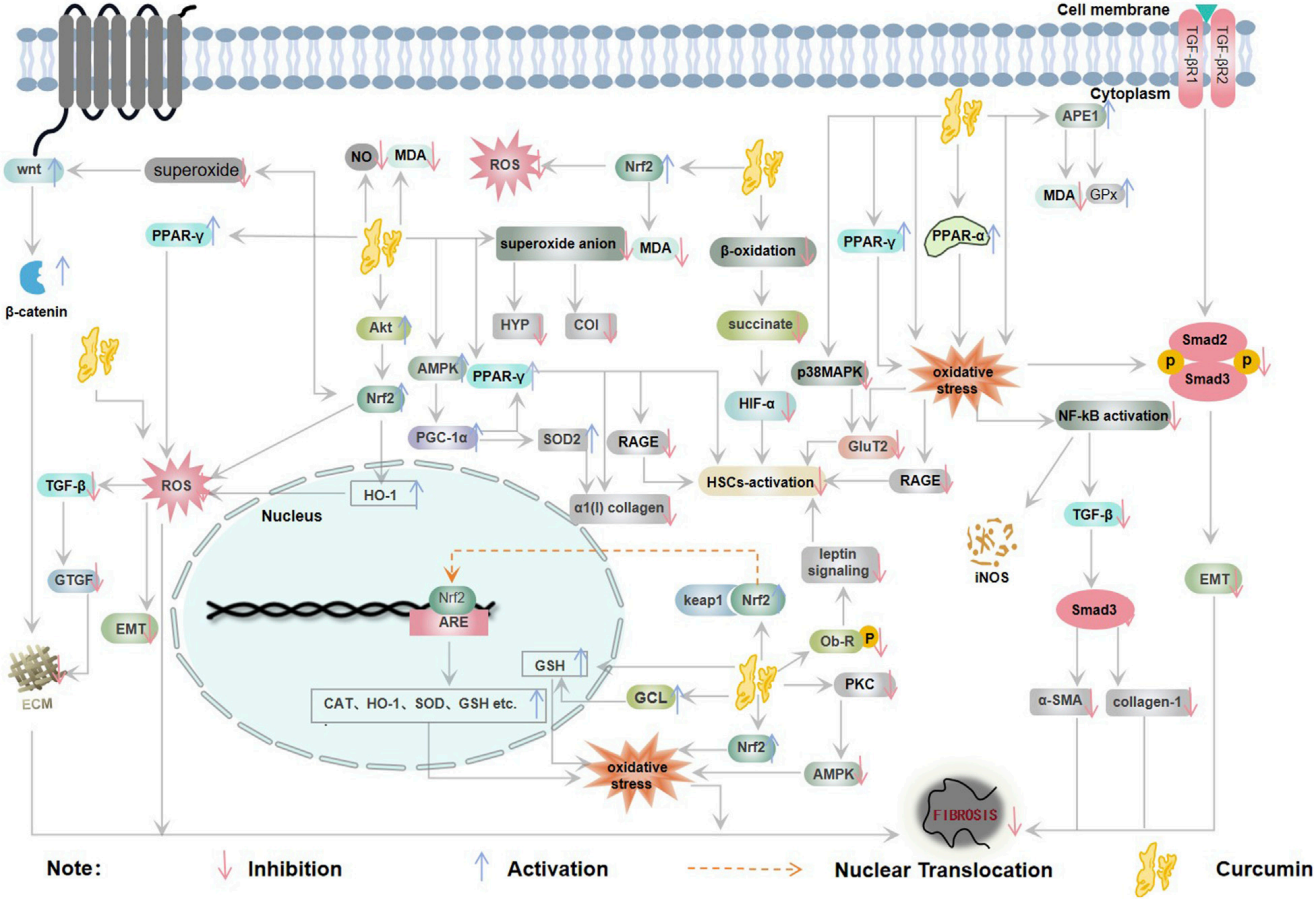
Relationship between oxidative stress and fibrosis (CAT: catalase, ECM: extracellular matrix, EMT: epithelial-mesenchymal transition, NO: nitric oxide, ROS: reactive oxygen species, SOD: superoxide dismutase).

In the pathological mechanism of oxidative stress-induced fibrosis, Oxidative stress drives inflammatory cascades via elevated secretion of proinflammatory cytokines and fibrogenic growth factors, thereby promoting myofibroblast activation and pathological extracellular matrix remodeling. ROS upregulates TGF-β signaling pathways and serve as critical effectors in propagating TGF-β-induced fibrogenic responses, including fibroblast activation, the synthesis of pro-fibrotic mediators, epithelial/endothelial cell apoptosis, and epithelial-mesenchymal transition (EMT) (Liu and Desai, 2015). On the other hand, increased extracellular matrix deposition due to oxidative stress subsequently leads to fibrosis (Jianming and Li, 2016; Makena et al., 2023). In the meanwhile, oxidative stress causes lipid damage to parenchymal cell membranes, disrupts enzyme and protein modifications that are essential for cell metabolism, and promotes DNA mutation, culminating in apoptosis of these cells. In summary, oxidative stress can cause the progress of fibrotic diseases through these damages (Antar et al., 2023) (Figure 2).

## 6 Effect of curcumin on fibrotic diseases

**FIGURE 3:**
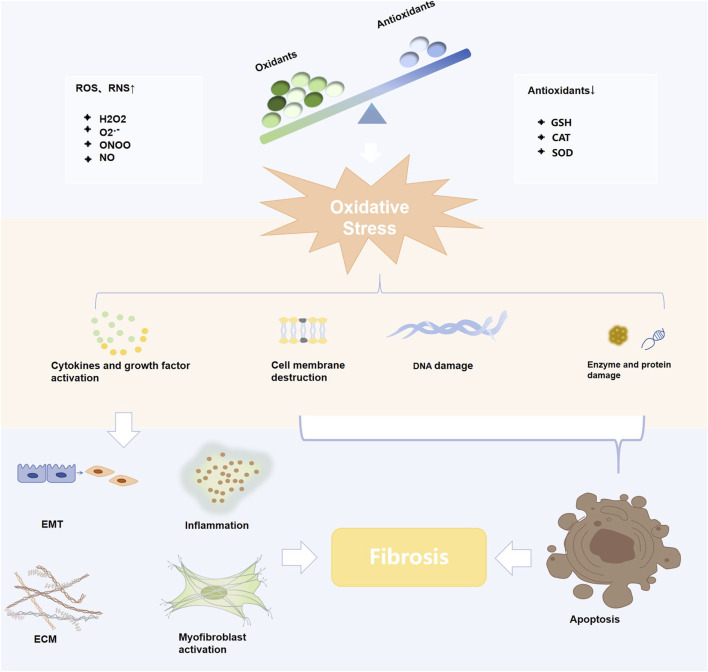
Diagram of the mechanisms of curcumin against various fibrotic diseases.

The present study systematically elucidates the multifaceted antifibrotic therapeutic mechanisms of curcumin (Figure 3).

### 6.1 Anti-hepatic fibrosis effect of curcumin

PPARα (NR1C1), a ligand-responsive nuclear hormone receptor, demonstrates pronounced hepatic tissue enrichment, was originally characterized as a pharmacological target for heterogenin that induces peroxisome proliferation in rodents (Issemann and Green, 1990). Besides PPARα, this receptor subclass includes two additional variants specified by the PPARβ/δ (NR1C2) and PPARγ (NR1C3) genes, with each exhibiting organ-specific expression profiles and distinct physiological roles (Kliewer et al., 1994). Hepatic PPARα serves as a master regulator of β-oxidation processes and systemic lipid/energy equilibrium (Janovick et al., 2022; Qiu et al., 2023). Nuclear receptor PPARα is predominantly activated under energy-deprived states, orchestrating mitochondrial energetic reprogramming that culminates in enhanced oxidative phosphorylation for ATP synthesis. Curcumin inhibits autophagy induced by oxidative damage in hepatic cells via PPAR-α activation, thereby reducing the occurrence of EMT, decreasing ROS and MDA, and inhibiting the production of ECM, thus playing a crucial part in improving hepatic fibrosis (Elmansi et al., 2017; Kong et al., 2020).

PPARγ serves as a differentiation marker for hepatic stellate cells (HSCs), with its transcriptional activity declining during their transition to myofibroblasts, whereas agonist-mediated inhibition suppresses HSC activation (Li et al., 2015). Curcumin upregulates PGC-1α via AMPK signaling, subsequently enhancing PPARγ activity and Superoxide Dismutase-2 (SOD-2) transcription/activity, thereby suppressing α1(I) collagen expression in cultured HSCs (Zhai et al., 2015). Within HSCs, curcumin enhances PPARγ functionality while mitigating oxidative stress through Ob-R dephosphorylation, subsequently suppressing Ob-R transcription and blocking leptin-mediated signaling, thereby abolishing leptin’s pro-activation effects on HSCs (Tang et al., 2009). It additionally suppressed receptor of advanced glycation endproducts (RAGE) transcriptional activity via enhanced PPARγ functionality and attenuated oxidative stress, thereby abolishing receptor of advanced glycation endproduct (AGE)-mediated stimulation of HSC activation (Lin et al., 2012). Furthermore, curcumin mitigates liver fibrosis progression through PPAR-γ activation, leading to elevated glutathione levels and diminished oxidative stress within activated hepatic stellate cells (Zheng and Chen, 2006).

GSH, serving as the primary intracellular redox buffer, demonstrates particularly high concentration within hepatic tissue (Vairetti et al., 2021). Curcumin can upregulate glutathione, GSH/GSSG ratio and total glutathione levels thus improving hepatic fibrosis in Wistar rats (Reyes-Gordillo et al., 2008; Hernández-Aquino et al., 2020). Research demonstrates curcumin suppresses GLUT2 transcriptional activity via PPARγ activation and promotes *de novo* glutathione biosynthesis, thereby eliminating HSC activation and ultimately ameliorating hyperglycemia-associated liver fibrosis (Lin and Chen, 2011).

Glutamate cysteine ligase (GCL) serves as a critical regulatory enzyme governing GSH biosynthesis. Curcumin induces GCL expression to increase GSH and reduce oxidative stress, thereby preventing liver fibrosis formation in HSCs and Sprague-Dawley rats (Zheng et al., 2007; Fu et al., 2008).

NRF2 mediates the expression of multiple genes and influences various physiological processes, including substance metabolism, ROS clearance, and glutathione synthesis (Hayes and Dinkova-Kostova, 2014). Curcumin can protect HSCs from oxidative stress by upregulating NRF2 and inhibiting the activation and secretion of glucose oxidase (Go) -induced ECM molecules (Liu et al., 2016; Gowifel et al., 2020).

Nitrotyrosine is a product of protein oxidation and is considered a marker of oxidative damage. Curcumin may prevent liver fibrosis through the decrease of nitrotyrosine staining in thioacetamide (TAA) -treated rats (Bruck et al., 2007). Overproduction of ROS oxidizes guanine residues to 8-OHdG, and these adducts can cause DNA damage when added to DNA (Singh et al., 2011). Curcumin significantly diminishes the proportion of 8-OH-dG-immunopositive nuclei, serving as an established biomarker for oxidative stress-induced DNA damage (Vizzutti et al., 2010). Heme oxygenase-1 (HO-1) serves as the primary regulatory enzyme in heme metabolism, exhibiting pronounced antioxidant and anti-inflammatory properties (Yachie, 2021). Curcumin may ameliorate hepatic fibrosis by increasing HO-1 and decreasing oxidative stress in Male Sprague–Dawley rats (Öner-İyidoğan et al., 2014). Apurinic/apyrimidinic endonuclease 1 (APE1) plays a crucial part in the base excision repair (BER) pathway of ROS-induced damaged bases and DNA single-strand breaks and is a key element of proteins upregulated by oxidative stress (Weaver et al., 2022). Curcumin may protect the liver from oxidative stress via upregulating APE1 (Bassiouny et al., 2011). AGEs and RAGE play a pivotal role in NASH- associated hepatic fibrosis (Lohwasser et al., 2009). Curcumin ameliorates oxidative stress-induced effects by suppressing AGEs-induced leptin signaling activation, thereby blocking HSC activation (Tang and Chen, 2014).

In addition, curcumin inhibits MDA formation and significantly improves liver antioxidant status (Wu et al., 2008). In N-(4-hydroxyphenyl) acetamide (NHPA) -treated rats, curcumin attenuated hepatic collagen III accumulation and fibrogenesis by inhibiting nitric oxide and MDA production. At the same time, curcumin can improve the levels of GSH, SOD, and CAT in liver fibrosis induced by thioacetamide and bisphenol a, and reduce MDA (Elswefy et al., 2020; Radwan et al., 2024). Abnormal accumulation of succinate can induce ROS production (Chouchani et al., 2014). Hypoxia inducible factor- 1α (HIF-1α) is involved in cell cycle change, extracellular matrix deposition, and myofibroblast transition (Senavirathna et al., 2018; Aquino-Gálvez et al., 2019). Curcumin prevents HSC upregulation by suppressing the succinic acid/HIF-1α signaling cascade and reducing succinic acid accumulation by countering fatty acid oxidation (She et al., 2018).

In summary, the anti-fibrotic action of curcumin in the liver is characterized by its multi-pronged attack on the hepatic triad of injury: it dampens Kupffer cell activation, directly suppresses HSC activation, and enhances hepatoprotective signaling. The targeting of resident liver cells like Kupffer cells and HSCs presents a distinct, liver-specific strategy not seen in other organs. This contrasts with its role in pulmonary fibrosis, where its primary cellular targets are likely activated fibroblasts and alveolar epithelial cells, highlighting how curcumin’s efficacy is shaped by the unique cellular ecosystem of each diseased organ.

### 6.2 Anti-renal fibrosis effect of curcumin

High glucose induces superoxide imbalance in glomerular mesangial cells and causes the accumulation of ECM in diabetic glomeruli. Renal tubular EMT is a factor in the accumulation of renal matrix proteins, and oxidative stress may predispose to the development of EMT in renal tubular epithelial cells in diabetic nephropathy. Evidence indicates curcumin ameliorates renal fibrosis via Wnt/β-catenin modulation coupled with superoxide suppression (Ho et al., 2016). It can also protect NRK-52E cells from high glucose-stimulated EMT by enabling NRF2 and HO-1 (Ho et al., 2016).

NRF2 is a cytoprotective transcription factor that can induce the expression of multiple antioxidants and phase II detoxification enzymes and plays an essential part in adjusting cellular detoxification and redox homeostasis. Evidence indicates curcumin ameliorates cisplatin-induced EMT and renal fibrosis via NRF2 activation (Trujillo et al., 2016). Soetikno et al. suggested that curcumin can also alleviate redox imbalance and renal fibrosis by governing the NRF2-Keap1 signaling cascade (Soetikno et al., 2013). Lia et al. suggested that curcumin could attenuate oxidative stress and renal fibrosis caused by acetaldehyde by activating the NRF2 signaling cascade to reduce MDA content and increase the levels of SOD, CAT, glutathione peroxidase (GPX), glutathione reductase (GR) and GSH (Li et al., 2019). Furthermore, it has also been suggested that curcumin can not only reverse the suppressive effect of redox imbalance on GPx efficacy, thereby alleviating the ROS accumulation in rats resulted by Ochratoxin A, but also alleviate the renal fibrosis caused by Ochratoxin A (Damiano et al., 2020).

The landscape of renal fibrosis underscores a central role for oxidative stress in driving mitochondrial dysfunction and epithelial-mesenchymal transition (EMT). Curcumin’s intervention in this organ demonstrates a distinctive emphasis on rectifying metabolic derangements within highly metabolic renal tubular cells. The significant amelioration of renal fibrosis through the Wnt/β-catenin and Nrf2-Keap1 pathways highlights a therapeutic strategy that is particularly crucial in the context of diabetic nephropathy. This metabolic-centric approach presents a contrast to its action in hepatic fibrosis, where the primary battleground involves the activation of quiescent hepatic stellate cells by inflammatory signals from Kupffer cells. Thus, in the kidney, curcumin appears to function not only as an antioxidant but also as a metabolic regulator, protecting the energy-fragile tubular epithelium from oxidative injury and subsequent fibrotic transformation.

### 6.3 Anti-myocardial fibrosis effect of curcumin

Cardiac fibrosis serves as a hallmark characteristic of pathological hypertrophy, manifesting as extracellular matrix proliferation driven by collagen deposition. Treatment of H9C2 cells with palmitate significantly increased ROS and redox imbalance, curcumin activated the NRF2 signaling cascade, thereby significantly increasing the expression of downstream genes glutamate-cysteine ligase catalytic (Gclc), HO-1, and NAD(P)H quinone oxidoreductase 1 (NQO-1), and antioxidant response inhibited myocardial fibrosis (Zeng et al., 2015). Studies have shown that the protein kinase C (PKC) is a serine/threonine-associated protein kinase (Newton, 2003). Hyperglycemia leads to PKC activation, and increased PKC activity can lead to alterations in the ECM (Sheetz and King, 2002), leading to cardiomyocyte hypertrophy and interstitial fibrosis, PKC activation can also induce mitogen-activated protein kinase (MAPK). Vivian Soetikno and Flori R. Sari et al. suggested that curcumin could ameliorate myocardial fibrosis in diabetic rats by suppressing the PKC-MAPK signaling cascade and attenuating oxidative stress (Soetikno et al., 2012). It is also reported that curcumin can reduce redox imbalance by activating the PPAR-γ pathway, thereby inhibiting inflammation and fibrosis (Gbr et al., 2021).

### 6.4 Anti-pulmonary fibrosis effect of curcumin

ROS/RNS produced either endogenous or extrinsic may damage alveolar epithelium directly (Kinnula et al., 2005). Oxidative stress occurred by activating transcription factors that trigger cell-mediated cell signaling pathways and induce inflammatory cytokines, while also damaging DNA and lipids (Bezerra et al., 2023; Liu et al., 2023). Research indicates that curcumin augments antioxidant capacity via upregulating HO-1 expression in fibroblasts and primary pulmonary endothelial cells while suppressing radiation-triggered (Lee et al., 2010). In murine LMSCs, curcumin may have an antioxidant effect on it through the Akt/Nrf2/HO-1 pathway, thereby playing a role in anti-pulmonary fibrosis (Ke et al., 2020). Animal experiments showed that curcumin inhibited hydroxyproline content, collagen type I, TGF-β1 expression, myeloperoxidase (MPO), and superoxide generation in the lungs of amiodarone rats, thereby improving pulmonary fibrosis (Punithavathi et al., 2003). According to previous studies that TGF-β1 can induce NADPH oxidase 4 (NOX4)-dependent ROS production, thereby promoting fibroblast migration (Amara et al., 2010), and can upregulate mitochondrial ROS to induce lung epithelial cell senescence to promote fibrosis (Yoon et al., 2005; Taslidere et al., 2014). Furthermore, curcumin and nanocurcumin inhibited ROS production and alleviated paraquat (PQ) -induced pulmonary fibrosis by modulating gene expression of the kelch-like ECH-associated protein 1 (KEAP1), HO-1, NQO1, and glutathione-S-transferase (GST) in lung tissue (Tyagi et al., 2016; Mahlooji et al., 2022). We summarized the effects of curcumin on various fibrotic diseases in Table 2 (Table 2).

**TABLE 2:** Effect of curcumin on fibrotic diseases.

Disease	Animals/cell lines	Upregulation	Downregulation	Inducers	Concentration	Duration	References
Pulmonary Fibrosis	Female C57BL/6 mice	—	HYP	RT	1% or 5% curcumin	4 months	Lee et al. (2010)
	PMVEC	—	ROS	RT	5, 10, 25, 50, 100 μM	4 h	
	Primary fibroblasts	—	—	—	5, 10, 25, 50, 100 μM	4 h	
	Murine LMSCs	p-Akt/Akt, NRF2, HO-1	ROS	H2O2	2.5, 5, 10 μM	6 h	Ke et al. (2020)
	Male Fischer 344 rats	—	MPO, HYP, Type I Collagen, Superoxide anion,TGF-β1	Amiodarone	200 mg/kg	5 weeks	Punithavathi et al. (2003)
	Parke’s strain of mice	—	HYP, ROS, TIMP-1, a-SMA	PQ	5 mg/kg	49 h	Tyagi et al. (2016)
	Female mice	—	ROS, α-SMA, HYP, Nitrite, MPO, EPO	SiO2	5 mg/kg	21 days	Kumari and Singh (2022)
	Male Wistar rats	NRF2, HO-1, NQO1, TAC, TTG, GST	KEAP1, Hydroxyproline	PQ	30 mg/kg/day	7 days	Hosseini et al. (2021)
Hepatic Fibrosis	Male Sprague-Dawley rats	—	HYP, HA, PC III, Collagen IV, α-SMA	CCl4	100, 200, 400 mg/kg	8 weeks	Kong et al. (2020)
	BNL CL.2 cells	PPAR-α, GSH	α-SMA, ROS	TGF-β1	10, 20, 30 μM/L	24 h	
	Male Wistar rats	GSH	α-SMA, Col-I, Smad3, CTGF, TGF-β	CCl4	100 mg/kg	4 weeks	Hernández-Aquino et al. (2020)
	Male ICR mice	—	α-SMA, HIF-1α, Col1α, Col3α, FN, TGF-β1, SDH, succinate	HFD	50 mg/kg	10 weeks	She et al. (2018)
	HSCs	—	Col1α, Col3α, FN, α-SMA, TGF-β1, ROS	dimethyl succinate	10 μM	8 h	
	HSCs	—	SDH, HIF-1α	PA	10 μM	8 h	
	Male Wistar rats	GSH, GSSG, GSH+GSSG, GSH/GSSG	collagen, TGF-β	CCl 4	100 mg/kg	2 months	Reyes-Gordillo et al. (2008)
	Male Wistar rats	—	Nitrotyrosine	TAA	300 mg/kg	12 weeks	Bruck et al. (2007)
	Male Sprague Dawely rats	SOD, HO-1	ROS, MDA	TAA	100, 200 mg/kg	18 weeks	Elmansi et al. (2017)
	Male C57BL/6 mice	—	α-SMA,TIMP-1, Procollagen type I, ROS, 8-OH-dG	MCD	25 μg	4, 8, 10 weeks	Vizzutti et al. (2010)
	Male Sprague–Dawley rats	SOD, HO-1	MDA, ROS	HFD	1 g/kg	16 weeks	Öner-İyidoğan et al. (2014)
	HSCs	PGC-1α, AMPKα, SOD2, PPAR-γ	α1(I) collagen	—	5, 10, 15 μm	24 h	Zhai et al. (2015)
	Sprague-Dawley rats	GSH, GCL, GSH/GSSG, PPARγ	α-SMA, αI(I) collagen, FN, Tβ-RII, Tβ-RI, Lipid hydroperoxide, HYP, PDGF, TGF-β	CCl4	200, 400 mg/kg	8 weeks	Fu et al. (2008)
	HSCs	PPAR-γ, GSH, GCL, GSH/GSSG	α-SMA, αI(I) collagen, TGF-βRI, TGF-βR II, ROS, LPO, Ob-R, PDGF-βR, CTGF	Leptin	5, 10, 20, 30 μM	24 h	Tang et al. (2009)
	HSCs	PPARγ, GSH, GSH/GSSG, GCL	p38 MAPK, αI(I) procollagen, α-SMA, Tβ-RI, Tβ-II, CTGF, ROS, LPO, GLUT2, PDGF-βR	Glucose	10, 20, 30 μM	24 h	Lin and Chen (2011)
	HSCs	PPAR-γ, GSH, GSH/GSSG, GCL	Tß-RI, Tß-RII, ROS, LPO, αI(I) collagen	—	5, 10, 15, 20, 30 µM	24 h	Zheng et al. (2007)
	Male Sprague-Dawley rats	GSH, GPX, APE1, PPARγ	MDA, TGF-β, CTGF, TIMP-1, α-SMA, STAP	CCl4	200 mg/kg	4 or 8 weeks	Bassiouny et al. (2011)
	HSCs	PPAR-γ, GSH, GSH/GSSG	Tβ-RI, Tβ-RII, CTGF, αI(I)-procollagen, α-SMA	—	20 μM	24 h	Zheng and Chen (2006)
	Male Sprague-Dawley rats	SOD, GSH	MDA	CCl4	0.005% curcumin in feed	8 weeks	Wu et al. (2008)
	Male Wistar rats	NRF2, SOD, GSH, HO-1	MPO, MDA, iNOS, Collagen type I, α-SMA, NF-ĸB-p65, TGF-β, p-Smad3	TAA	200 mg/kg	8 weeks	Gowifel et al. (2020)
	HSCs	PPARγ, GSH, GSH/GSSG, GCL	RAGE, ROS, LPO	AGEs	5, 10, 20, 25, 30 µM	24 h	Lin et al. (2012)
	HSCs	NRF2, GCL, GSH, AGE-R1	Leptin, RAGE	AGEs	20 mM	—	Tang and Chen (2014)
	Male Wistar albino rats	CAT, GSH, TIMP-2	MDA	BPA	100 mg/kg	8 weeks	Elswefy et al. (2020)
	HSC-T6	NRF2, GSH	ROS, MDA, α-SMA	GO	0.15 µM	3 h	Liu et al. (2016)
	Male Wistar albino rats	SOD	NO, MDA, α-SMA, Collagen III	NHPA	200 mg/kg	22 h	Alhusain et al. (2022)
	Male Rattus norvigicus	CAT, SOD, GSH	MDA	TAA	Curcumin group:50 mg/kg; Curcumin NPs group:15 mg/kg	2 weeks	Radwan et al. (2024)
Renal Fibrosis	Male Wistar rats	Wnt5a, β-catenin	Superoxide, 8-OH-dG, Fibronectin, TGF-β1	STZ	10 mg/kg/day	56 days	Ho et al. (2016)
	Rat mesangial cells	Wnt5a, β-catenin	Superoxide, TGF-β1, Fibronectin	D-glucose	10 μM	48 h	
	Male Wistar rats	NRF2, CAT, GR	TGF β1, Collagen I, Collagen IV, a-SMA, MDA, 3-NT, p47phox, gp91phox, PKCβ2	CIS	200 mg/kg	72 h	Trujillo et al. (2016)
	The NRK-52E normal rat/kidney tubular epithelial cell line	NRF2, HO-1	α-SMA	HG	5, 10, 20 µM	24 h	Zhang et al. (2015)
	Male Sprague-Dawley rats	NRF2, HO-1, GPx, CCr	Keap1, p67phox, p22phox, MDA, NF-κB, TNF-α, TGF-β1, Fibronectin	5/6 nephrectomy	75 mg/kg/day	8 weeks	Soetikno et al. (2013)
	Male Sprague Dawley rats	SOD, CAT, Gpx	MDA	OTA	100 mg/kg	14 days	Damiano et al. (2020)
	Male C57BL/6 mice	GSH, NRF2, HO-1, NQO1, UGT, SOD, CAT, GPx, GR	MDA, α-SMA, Collagen I	Glyoxylate	50,100 mg/kg	14 days	Li et al. (2019)
Myocardial Fibrosis	Male adult Sprague Dawley rats	GSH, TAC, PPAR-γ	MDA, TGF-β1, CaMKII	STZ	100 mg/kg/day	6 weeks	Gbr et al. (2021)
	Male C57BL/6 mice	NRF2, HO-1, NQO-1	CTGF, TGF-β	HFD	50 mg/kg/day	8 weeks	Zeng et al. (2015)
	H9C2 embryonic rat heart-derived cell line	NRF2, HO-1, GCLC, NQO-1	ROS, TGF-β	PA	20 μM	15 h	
	Male Sprague–Dawley rats	GPx	MDA, p22phox, p67phox, gp91phox, PKC-α, PKC-β2, p-P38MAPK/P38MAPK, p-ERK1/2/ERK1/2, TGF-β	STZ	100 mg/kg/day	8 weeks	Soetikno et al. (2012)

The evidence presented above positions curcumin as a modulator of the oxidative milieu that drives fibroblast differentiation and ECM deposition in the lungs. A key comparative insight emerges when contrasting lung and liver fibrosis: while both conditions involve TGF-β signaling, the upstream triggers and key effector cells differ substantially. In the liver, curcumin’s interception of damage signals from hepatocytes to Kupffer cells and HSCs is critical. In the lung, however, its ability to mitigate epithelial cell injury and its consequent signaling to fibroblasts may be of paramount importance. This underlines the concept that while core pathways like Nrf2 and TGF-β are shared therapeutic targets, the cellular “entry point” for curcumin’s action is organ-specific.

### 6.5 Anti-fibrotic effect of curcumin analogues and formulations

**FIGURE 4:**
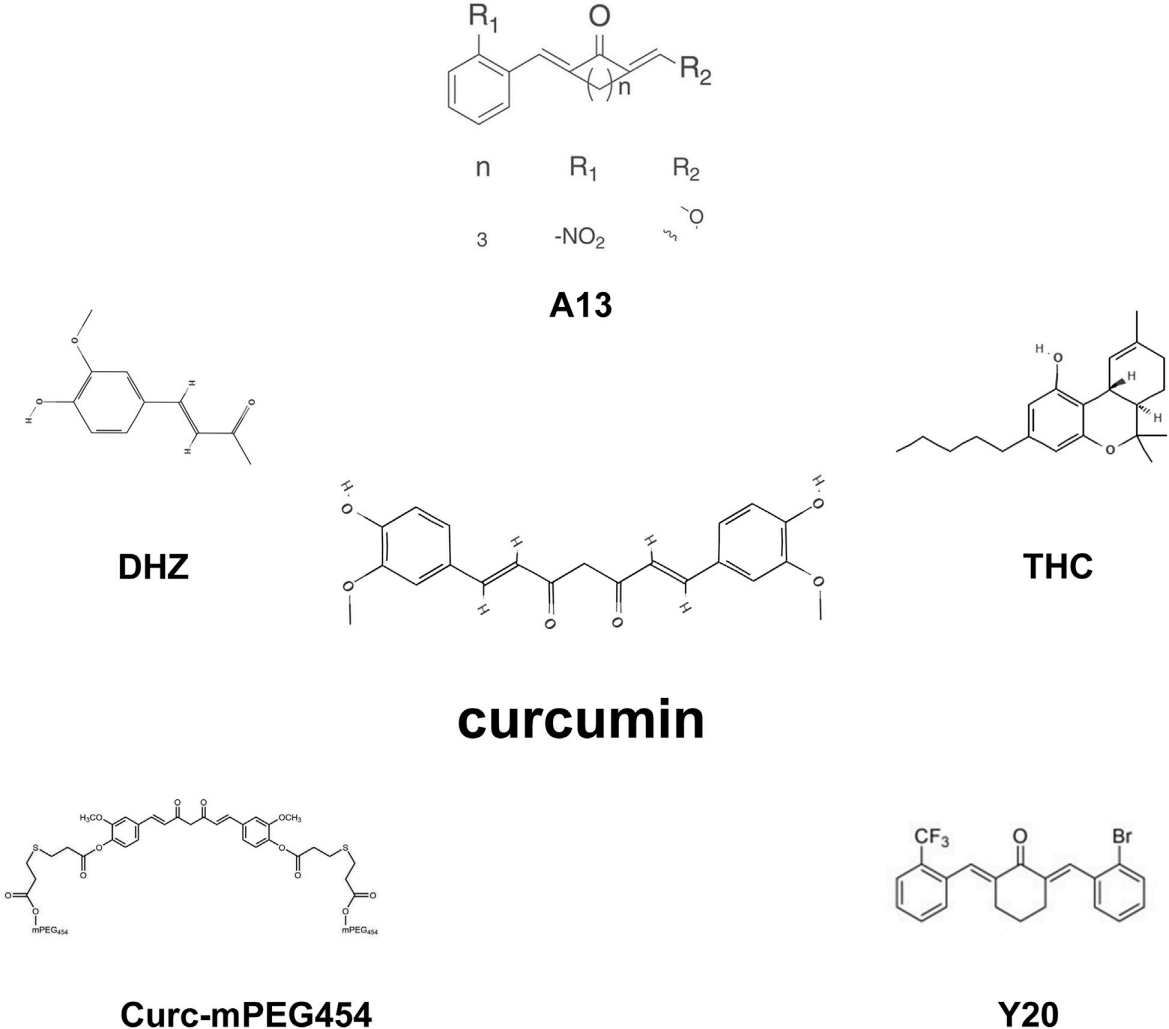
The molecular structures of the curcumin analogues.

Removal of unstable molecular groups and retention of active molecular groups by curcumin analogues can enhance their instability. A13, classified among curcumin analogues, shares fundamental pharmacological properties with curcumin yet demonstrates enhanced efficacy compared to curcumin regarding metabolic stability and oral bioavailability. It reduces oxidative stress and improves myocardial fibrosis in diabetic rats by activating the Nrf2/ARE pathway (Xiang et al., 2020). In addition, curcumin analog Y20 has shown better pharmacokinetic profile *in vivo* and can exert dual anti-inflammatory and antioxidant activities. It activates Nrf2 expression and thereby regulates oxidative stress, which may mediate high-fat diet-induced cardiomegaly, apoptosis, and fibrosis (Qian et al., 2015). Curc-mPEG454, a curcumin conjugate functionalized with short-chain polyethylene glycol (PEG), demonstrates elevated serum concentrations of curcumin while preserving its anti-inflammatory efficacy. Curc-mPEG454 augments cellular redox homeostasis through stimulation of *de novo* biosynthesis of Nrf2-mediated GSH (Xiao et al., 2021). The main metabolite of curcumin is tetrahydrocurcumin (THC), which is superior in inducing glutathione peroxidase and quenching free radicals, and is more stable and has better intestinal absorption than curcumin. Dietary tetrahydrocurcumin can improve renal fibrosis by reducing copper-zinc superoxide dismutase (CuZn SOD) and glutathione peroxidase (GPX-1) (Lau et al., 2018). Dehydrogingerone (DHZ), a polyphenolic constituent isolated from the rhizome of Zingiber officinale, which is a semi-analog of curcumin. DHZ ameliorated the TAA-mediated downregulation of catalase activity, thereby alleviating hepatic fibrosis progression (Sharma et al., 2022). C66, a recently developed curcumin analogue, exhibits a significantly reduced effective dosage. It stimulates miR-200a, downregulates Keap1 expression, activates NRF2, alleviates redox imbalance, and thus anti-renal fibrosis (Wu et al., 2016). Curcumin nanoparticles can improve the bioavailability and biodistribution, which significantly decreased MDA and significantly improved CAT, SOD and GSH in thioacetamide (TAA) -stimulated hepatic fibrosis in rats (Radwan et al., 2024) (Figure 4). These structural and delivery innovations directly address curcumin’s pharmacokinetic limitations. A13 and C66 analogues enable lower dosing in fibrosis models, while nanoformulations like Curc-mPEG454 achieve sustained plasma exposure critical for chronic fibrosis management. This paves the way for human trials targeting organ-specific accumulation.

### 6.6 Transcriptional reprogramming of fibrotic pathways by curcumin

Curcumin orchestrates transcriptional suppression of fibrotic genes through interconnected mechanisms. In hepatic fibrosis models, it inhibits succinate accumulation by blocking succinate dehydrogenase activity, thereby preventing HIF-1α-mediated upregulation of Col1α, Col3α (She et al., 2018). Simultaneously, it reprograms AGE receptor expression in hepatic stellate cells—downregulating pro-fibrotic RAGE while upregulating detoxifying AGE-R1—via interruption of leptin signaling and Nrf2 activation (Tang and Chen, 2014). Furthermore, curcumin (200 mg/kg) induces APE1 expression in fibrotic livers, enhancing DNA repair while suppressing NF-κB-driven transcription of TNF-α and IL-6 (Bassiouny et al., 2011).

## 7 Clinical evidences supporting the anti-fibrotic effects of curcumin

In patients with non-alcoholic fatty liver disease (NAFLD), a randomized controlled trial (RCT) found that 12 weeks of curcumin supplementation (1,500 mg/day) significantly reduced hepatic fibrosis scores compared to baseline, although it was not superior to lifestyle modification alone in ameliorating systemic inflammation (Saadati et al., 2019). The disparity in outcomes underscores the potential need for longer intervention periods or more bioavailable formulations, a notion strongly supported by a 12-month RCT in patients with metabolic dysfunction-associated steatotic liver disease (MASLD) and type 2 diabetes, where 1,500 mg/day of curcumin led to significant reductions in both hepatic steatosis and liver stiffness, alongside marked improvements in systemic inflammation and oxidative stress (Yaikwawong et al., 2025).

The most compelling evidence for curcumin’s direct anti-fibrotic action in human tissue comes from a 72-week, double-blind RCT using the phospholipid formulation Meriva (2 g/day) in patients with biopsy-proven non-alcoholic steatohepatitis (NASH) (Musso et al., 2025). This study reported that a remarkable 42% of patients on Meriva achieved regression of significant liver fibrosis, with 62% experiencing NASH resolution, effects potentially mediated by the inhibition of hepatic NF-κB. Importantly, the same trial also observed a significant regression of concomitant chronic kidney disease in 50% of the Meriva group, suggesting systemic anti-fibrotic benefits (Musso et al., 2025). To specifically validate these findings in renal fibrosis, the large-scale, multicenter MPAC-CKD-1 trial was designed to evaluate the efficacy of a micro-particle curcumin formulation on albuminuria and estimated glomerular filtration rate (eGFR) in patients with chronic kidney disease, with results anticipated to provide crucial evidence on its potential to slow disease progression (Weir et al., 2018).

## 8 Future perspectives

Curcumin exhibits a broad spectrum of positive effects in mediating oxidative stress for therapeutic intervention in fibrotic diseases. In the present investigation, the molecular mechanism of curcumin-mediated oxidative stress against fibrotic diseases was reviewed, such as PPAR, NRF2, HO-1, TGF-β, AGE-RAGE, and other signaling pathways. Curcumin functions as a free radical scavenger through its phenolic substances, beta-diones, and methoxys, acting on the activity of ROS (Farzaei et al., 2018). In liver fibrosis, damage to liver cells causes the formation of apoptotic bodies (AB), the leakage of mitochondrial DNA, or the generation of ROS. These factors initiate the stimulation of Kupffer cells and the conversion of dormant hepatic stellate cells into myofibroblastic phenotypes. Leptin, Angiotensin II, TGF-β, Platelet-derived growth factor (PDGF), and other mediators help activate NOXs in macrophages and stellate cells and further accelerate matrix deposition. Reaserches have found that curcumin has shown prophylactic and curative effects on oxidization-related liver disease through multiple cells signaling cascades. These pathways include upregulated PPAR (Zhai et al., 2015; Elmansi et al., 2017; Kong et al., 2020), NRF2 (Liu et al., 2016; Gowifel et al., 2020) and HO-1 (Öner-İyidoğan et al., 2014) signaling pathways, and downregulated RAGE (Lin et al., 2012) and AGEs (Tang and Chen, 2014) signaling pathways. In pulmonary fibrosis, ROS generation contributes to fibroblast phenotype acquisition, including differentiation, contraction, apoptotic resistance, and ECM deposition (Hecker et al., 2009; Hosseinzadeh et al., 2018). Evidence suggests that curcumin can activate the protein kinase B (Akt)/Nrf2/HO-1 (Lee et al., 2010; Ke et al., 2020) signaling pathway and attenuate the TGF-β1, MPO (Li et al., 2019) and ROS (Kumari and Singh, 2022) to inhibit redox imbalance in pulmonary fibrosis models. In myocardial fibrosis, the cardiac source of ROS is mainly nicotinamide-adenine dinucleotide phosphate (NADPH) oxidase (Li et al., 2002), which exacerbates cardiac fibrosis and modulates gap junction function, resulting in diminished myocyte coupling and facilitating arrhythmogenic reentry (Sovari, 2016). Curcumin reduces cardiac fibrosis by activating SIRT1, increasing NRF2 (Zeng et al., 2015), increasing NADPH oxidase subunits, weakening PKC-MAPK (Soetikno et al., 2012) signaling pathway, and reducing SOD and MDA (Sadoughi et al., 2021). In renal fibrosis, redox imbalance can cause the reduction of ATP production after mitochondrial dysfunction, and may also lead to the development of EMT in diabetic nephropathy, resulting in renal fibrosis (Lv et al., 2018). Emerging evidence indicates that curcumin attenuates renal fibrosis through Wnt/β-catenin (Ho et al., 2016), HO-1 (Zhang et al., 2015) and Nrf2-Keap1 (Soetikno et al., 2013) signaling pathways. These provide references for the pharmacology and clinical application of curcumin for therapeutic intervention in fibrotic diseases. However, there are some questions that need to be further clarified in future studies before this natural compound can be used clinically.

Synthesizing the evidence from liver, renal, myocardial, and pulmonary fibrosis, a coherent model for curcumin’s pleiotropic actions comes into focus. We propose a “Core Pathway - Specific Branch” model to conceptualize its effects. At the heart of this model lies the consistent activation of the Nrf2 antioxidant pathway and the concerted suppression of the TGF-β signaling axis. These two core pathways, addressing the universal pillars of oxidative stress and pro-fibrotic signaling, form the foundational mechanism of curcumin’s efficacy across all organs studied.

Firstly, human pharmacokinetics of curcumin remain incompletely characterized, with low absorption and fast metabolism (Mirzaei et al., 2017). Secondly, limited absorption of curcumin severely constrains its clinical utility. Fortunately, chemical modification of curcumin significantly enhances its therapeutic efficacy, target selectivity, and safety profile. In addition to this, sophisticated drug delivery platforms, including liposomes, nanoparticles, and phospholipid complexes formulated with diverse synthetic/natural biomaterials (proteins, lipids, polymers), have demonstrated enhanced bioavailability and formulation stability for curcumin derivatives. The promising work on phospholipid complexes and nanoparticles must be advanced towards “smart” targeted delivery. Strategies should include designing nanoparticles that home to activated stellate cells or profibrotic fibroblasts, engineered for stimulus-responsive release in the high-ROS microenvironment of the fibrotic niche. This would ensure precise spatiotemporal delivery, enhancing efficacy while minimizing off-target effects. Combining curcumin with established anti-fibrotic drugs in a single nano-formulation could also create powerful synergistic therapies, where curcumin acts as a “sensitizing” agent to enhance the primary drug’s efficacy. Although extensive research efforts have addressed formulation limitations and optimized physicochemical attributes, critical gaps persist in curcumin’s therapeutic efficacy, target specificity, and pharmacokinetic performance—issues that warrant urgent attention from the scientific community (Bisht et al., 2007). Third, due to the multi-target properties of curcumin, its anti-fibrosis mechanism has not been fully illustrated and more validation is needed. The majority of contemporary investigations primarily emphasize cellular and animal-based models, yet scarce clinical evidence exists to assess curcumin’s anti-fibrotic therapeutic potential and its corresponding dosage requirements. More studies should provide more conclusive evidence, especially those with large samples and multi-center prospective cohort studies. The most critical frontier is the design of definitive clinical trials. The scarcity of clinical evidence, highlighted earlier, must be addressed through hypothesis-driven, biomarker-enriched trials. These trials should incorporate. Validated Redox and ECM Biomarkers: Moving beyond standard serum enzymes to include direct markers of oxidative stress (e.g., specific lipid peroxidation adducts) and ECM turnover (e.g., PRO-C3) to objectively quantify anti-fibrotic efficacy. Precision Enrollment: Focusing on specific fibrotic disease etiologies and potentially stratifying patients based on their baseline redox or inflammatory status. Long-term Safety and Efficacy Assessment: Rigorously evaluating the long-term safety profile, a concern we previously raised, and the ability of curcumin to halt or reverse fibrosis progression in large-scale, multi-center, randomized controlled trials. Fourth, concerning safety evaluation, extended-duration human studies are required to rigorously evaluate the therapeutic safety profile of curcumin in clinical populations.

## 9 Conclusion

In summary, numerous investigations have validated that the anti-fibrotic effect of curcumin through mediating oxidative stress, and more studies are needed to further confirm the anti-fibrotic effect of curcumin. It is hoped that with further research, the therapeutic effect of curcumin on fibrotic diseases may be understood and applied clinically.
